# HIV Preventive Behaviors and Associated Factors among Gold Mining Workers in Dima District, Southwest Ethiopia, 2019: Community-Based Cross-Sectional Study

**DOI:** 10.1155/2021/4797590

**Published:** 2021-10-18

**Authors:** Tadesse Nigussie, Yitagesu Mamo, Qaro Qanche, Tewodros Yosef, Wondimagegn Wondimu, Adane Asefa

**Affiliations:** ^1^Department of Reproductive Health and Nutrition, School of Public Health, College of Medicine and Health Sciences, Mizan Tepi University, Mizan-Aman, Ethiopia; ^2^Department of Clinical Pharmacy and Pharmacy Practice, School of Pharmacy, College of Medicine and Health Sciences, Mizan Tepi University, Mizan-Aman, Ethiopia; ^3^Department of Public Health, School of Public Health, College of Medicine and Health Sciences, Mizan Tepi University, Mizan-Aman, Ethiopia; ^4^Department of Epidemiology and Biostatistics, School of Public Health, College of Medicine and Health Sciences, Mizan Tepi University, Mizan-Aman, Ethiopia

## Abstract

**Background:**

HIV/AIDS is becoming more prevalent over time, resulting in a considerable number of deaths. In 2017, 36.9 million (35.1 million adults) people worldwide were living with HIV, 1.8 million people were newly infected with HIV, and 940 000 people died from AIDS-related illnesses. Mining workers are at a high risk of contracting HIV and infecting others, and effective prevention is a critical.

**Objective:**

To assess HIV preventive behaviors and associated factors among gold mining workers in Dima district, southwest Ethiopia, 2019.

**Methods and Materials:**

A community-based cross-sectional study was conducted from November 1^st^ to 30^th^, 2019. A convenience sampling technique was used to get mining workers for the interview.The data were collected through face-to-face interviews. The collected data were coded and entered into EpiData version 4.2.0.101, cleaned, and analyzed using SPSS version 21 statistical software. A binary logistic regression was conducted to determine the association using odds ratios at 95% confidence intervals. A *P* value of less than 0.05 considered the level of significance for HIV preventive behaviors.

**Results:**

From a total of 455 mining worker, 279 (61.3%) of them have good practices of HIV prevention. Factors associated with good preventive practice were not alcohol drinkers (AOR = 2.86, 95% CI: 1.30-6.29), not chew khat (AOR = 2.09, 95% CI: 1.09-4.02), having good knowledge about HIV (AOR = 1.81, CI: 1.16-2.83), favorable attitude towards HIV prevention (AOR = 4.76, 95% CI: 3.02-7.49), and high perceived susceptibility to HIV (AOR = 2.63, 95% CI: 1.63-4.24).

**Conclusion:**

Only about 61% of the mining workers in the study area were practiced HIV preventive behaviors. Not alcohol drinkers, having good knowledge about HIV, having a favorable attitude toward HIV prevention, and having high perceived susceptibility to HIV were factors associated with the practice of HIV preventive behaviors. Efforts have to be made by local governments and other concerned bodies to increase preventive behavior.

## 1. Introduction

The prevalence of HIV/AIDS is increasing over time, leading to a significant number of life losses [1, 2]. HIV epidemics continue to represent a public health threat worldwide, leading to causing deaths in Sub-Saharan Africa [3]. According to the USAID 2018 report, 36.9 million (35.1 million adults) people globally were living with HIV, 1.8 million people became newly infected with HIV, 940 000 people died from AIDS-related illnesses in 2017. Most at-risk populations (MARPs) and their sexual partners account for 47% of new HIV infections globally and 16% of new HIV infections in eastern and southern Africa. The risk of acquiring HIV is 13 times higher among female sex workers [1]. There is a high prevalence of HIV and other STIs in communities around goldmines [4]. Mineworker and women with a miner as a partner are most vulnerable to HIV/AIDS. Being a miner aged 30-44 years old increases the likelihood of being HIV positive by 15 percentage points in different countries. In addition to this, having a partner employed in the mines increases the probability of infection for women by 8 percentage points [5].

The adult HIV prevalence in Ethiopia in 2016 was estimated to be 1.1%. There is substantial prevalence variation by region (6.6% in Gambella, 5.0% in Addis Ababa, and 0.7% in Southern Nations, Nationalities and Peoples' (SNNPR) region) of Ethiopia [2]. According to Ethiopia Public Health Institute 2017 report, the prevalence of HIV shows a significant increment in adults of both sexes, but the incidence shows slow decrement. The annual AIDS-related deaths become decline from time to time [6].

Considering the fatal increment of HIV prevalence, there is a great struggle globally to end its epidemic. In 2014, UNAIDS launched new targets named 90-90-90 to help end the AIDS epidemic. According to this new target, by 2020, 90% of all people living with HIV will know their HIV status, 90% of all people with diagnosed HIV infection will receive sustained ART, and 90% of all people receiving ART will have viral suppression. In 2017, the global achievement of these three 90s was 75%, 79%, and 81%, respectively, which shows the need for extra effects for the full achievements [1, 7]. HIV testing is among the effective preventive activities. It can be the provider-initiated, voluntary-based, or self-test approach. It is being implemented in a major segment of the population by paying due attention to key populations like sex and mining workers [8–10]. The numbers of HIV testing among people in Sub-Saharan Africa have increased by 66% in the past decade and about one-third are diagnosed late, which can result in an increase in the risk of HIV-related morbidity and also can result in onwards transmission of HIV in the community [11].

Mining workers are among a population classified as MARPS. This is to indicate that the risk occurrence of HIV infection among this population is high. To control the HIV in general population, controlling the disease in this population is a key issue [10, 12–15]. For the successful control, evidence on prevalence rate, the practice of prevention methods, and experience of key populations are important.

The factors that contribute to the high prevalence of HIV/AIDS are diverse and might include education, income, female labor force participation, place of residence, demographic and cultural factors, male circumcision, condom use, access to counseling and testing, knowledge and awareness of HIV/AIDS virus, and access to antiretroviral therapy [16–18].

HIV prevention is a complex issue with no magic bullet for its success [19]. However, having good knowledge, attitudes, and practices (KAP) of HIV prevention is essential in order not to acquire HIV infection and to prevent the disease from spreading [11]. Adults and mining workers are the highest risk populations in acquiring HIV/AIDS when compared to others [8]. An improved knowledge, change attitude, and change behavior are considered the main factors that increase HIV test uptake [20]. So, this study was aimed to assess HIV preventive behaviors among mining workers in Dima woreda, southwest Ethiopia, 2019.

## 2. Methods and Materials

### 2.1. Study Area and Period

The study was conducted in Dima district, Gambella region southwest Ethiopia from November 1^st^ to 30^st^, 2019. Crop production is the main livelihood in Gambella where more 80% of the population is engaged in cereal production (mainly maize and sorghum). The market operates every day for both food/nonfood and livestock. Food and nonfood markets operate from 7:00 am to 5:00 pm; while the livestock market operates from 9:00 am to noon. For infrastructure, the market comprises a small, open market with temporary sheds and a livestock market place. Dima woreda is one of the woredas in Agnuak Zone, Gambela region of Ethiopia. The Dima district is among HIV high prevalent areas in Ethiopia. There are several miners and commercial sex workers in the area. There are about ten mining centers in the district.

### 2.2. Study Design

A community-based cross-sectional study was conducted among mining workers in Dima woreda.

### 2.3. Population

The source population were all mining workers in Dima district while the study population were randomly selected mining workers in Dima woreda.

### 2.4. Eligibility Criteria

Adults with age ≥ 18 were included in this study. Adults who fulfill the inclusion criteria but who are severely ill or unable to verbally communicate during the data collection time were excluded from the study.

### 2.5. Sample Size Determination and Sampling Technique

The sample size was determined by using the single population proportion formula considering the following assumptions: *p* = 50 percent (the proportion of good preventive behaviors); *d* = 0.05 margin of error, and 95% confidence level with a value of *Z* = 1.96; *n* = (*Z* *α*/2)^2^ *P* (1 − *P*)/*d*^2^ = 384. Using a nonresponse rate of 20% which give a final sample size of 461.

Regarding the sampling procedure, there are 10 rural kebeles in Dima district where mining workers were mostly found. We have randomly selected 5 kebeles, and the sample size was proportionally allocated for each of them, and a convenience sampling technique was used to get mining workers for interview.

### 2.6. Study Variables

#### 2.6.1. Dependent Variable

The dependent variable is the HIV preventive behaviors.

#### 2.6.2. Independent Variables

Independent variables include sociodemographic characteristics (age, sex, marital status, occupation, educational status, and income), cultural factors, and lifestyle factors and knowledge of HIV and its revention.

### 2.7. Data Collection Tools and Procedures

A structured questionnaire was developed from different works of literature [21–24]. The questionnaire has parts like sociodemographic, behavioural profile, knowledge of HIV, practices of HIV preventive beahaviours, and perceptions of HIV. Questionnaires were translated to Amharic from the English version and then back to English by an independent person to assure its accuracy. The questionnaire was pretested on 5% of the total sample size, which is a similar population to the study area Bero district. Bero is the nearer district with numbers of mining sites. The collected data were evaluated for completeness, clarity, and consistency by the supervisor and principal investigator on a daily basis. Ten data collectors and three supervisors who were qualified with bachelor of science (BSc) in Nursing were recruited and trained for two days before a data collection on data collection tool, approach to the interviewees, details of interviewing techniques, respect and maintaining privacy, and confidentiality of the respondents.

### 2.8. Data Processing and Analysis

The collected data were coded and entered using EpiData manager version 4.0.2.101, cleaned, and analyzed using SPSS version 21 statistical software. Summary statistics for different variables were presented using frequency tables and graphs. A binary logistic regression was computed to determine the association using crude and adjusted odds ratios at 95% confidence intervals. Independent variables with *p* values less than 25% were a candidate for multivariable logistic regression. A *p* value of less than 0.05 will be considered the level of significance for HIV preventive behaviours in the multivariable logistic regression.

### 2.9. Measurement and Operational Definitions

#### 2.9.1. Knowledge

It was measured by 17 yes or no questions. Then, the mean score was computed, and participants who scored greater than the mean score of knowledge questions were categorized as knowledgeable and not knowledgeable [25].

#### 2.9.2. Attitude

Assessed by 20 Likert questions ranging from 1 to 5 (strongly disagree to strongly agree), the negative questions were reverse coded and mean score was computed. Participants who scored greater than the mean score of attitude questions were categorized as having a positive attitude and having a negative attitude otherwise [26].

#### 2.9.3. Perceived Severity

Assessed by 6 Likert question ranging from 1 to 5 (strongly disagree to strongly agree), the negative questions were reverse coded and mean score was computed. Participants who scored greater than the mean score were categorized as having a high perceived severity, otherwise low [27].

#### 2.9.4. Perceived Susceptibility

Assessed by 6 Likert question ranging from 1 to 5 (strongly disagree to strongly agree), the negative questions were reverse coded and mean score was computed. Participants who scored greater thanthe mean score of attitude questions were categorized as having a high perceived susceptibility, otherwise low [27].

HIV preventive behavior is as follows: if an individual is abstaining from sexual intercourse in the last six months until the time of study period or having only one sexual partner and tested for HIV before their first sexual relationship, tested for HIV infection in last three month of the study period and consistently use a condom it was said to be in HIV preventive behavior [28].

## 3. Results

### 3.1. Sociodemographic Characteristics of Mining Workers

A total of 455 respondents participated giving a response rate of 98.6%. Majority of the respondents, 220 (48.4%), were in the age group of 25-34 years. The mean age of the participant was 25.84 (±5.34 SD). Three hundred (65.9%) of the respondents were single in marital status, 274 (60.8%) were orthodox Christianity followers, and 126 (27.7%) of the respondents were Amhara in ethinicity. One hundred fifty (33%) of the respondents completed secondary school. The median monthly income of respondents was 1000 Ethiopian birr, and 256 (56.3%) of them were earning less than 1000 birr (Table 1).

### 3.2. Knowledge about HIV Prevention and the Perception of HIV among Mining Workers

Four hundred twenty-eight (94.1%) of the respondents mentioned abstinence as the primary measure of HIV prevention. Being faithful was listed by 346 (76.0%). From total respondents, 226 (49.9%) of them had good knowledge regarding HIV prevention methods (Table 2).

### 3.3. Sources of Information about HIV and Its Prevention

**Figure 1:**
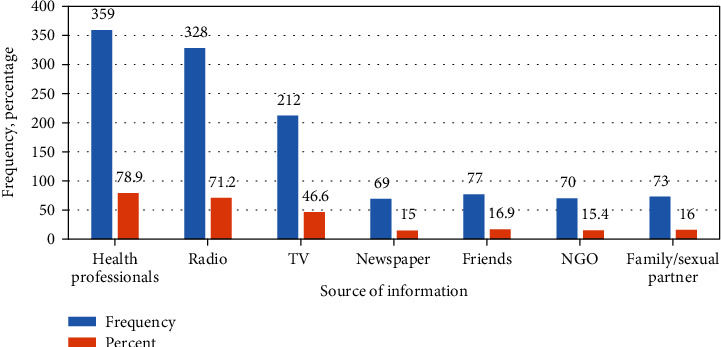
Sources of information about HIV and its prevention among mining workers in Dima district Gambella region southwest Ethiopia, November 2019.

The study participants have access to different sources of information on HIV, and the major source of information about the virus was health professionals (Figure 1).

### 3.4. Practices of HIV Prevention among Mining Workers

From interviewed participants, 415 (91.2%) were sexually active. Two hundred seventy-nine (61.3%) of the respondents have practices of HIV prevention. Three hundred eighty-nine (94.6%) of the respondents were sexually active in the last 12 months. Two hundred thirty five (57.3%) and 82 (20%) of the respondents had sexual intercourses with casual partners and with commercial sex workers, respectively. Two hundred twenty-one respondents (69.5%) had 2 and more sexual partners. Only 282 (81.3%) of them were using a condom consistently (Table 3).

### 3.5. Reason for Nonconsistent Condom Use

**Figure 2:**
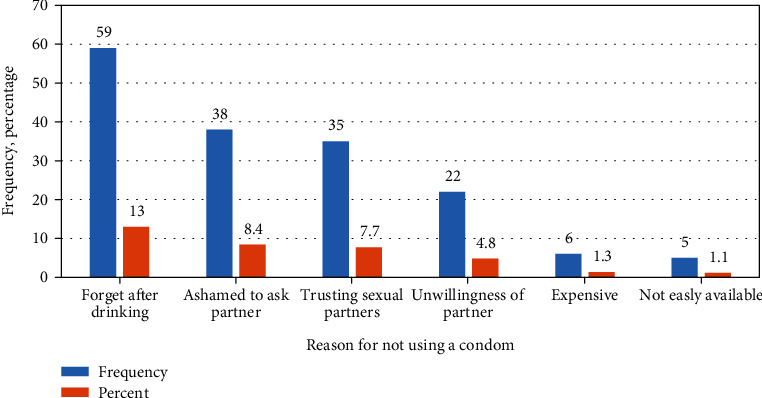
Reason of not using a condom consistently among mining workers in Dima district Gambella region southwest Ethiopia, November 2019.

The participating mining worker mentioned different reasons for a nonconsistent habit of condom use which may be risky for HIV transmissions. The most commonly listed reason for nonconsistent condom use was forgetfulness after drinking alcohol and being ashamed to ask partner for using condom (Figure 2).

### 3.6. Reason for Not Undergoing HIV Testing

**Figure 3:**
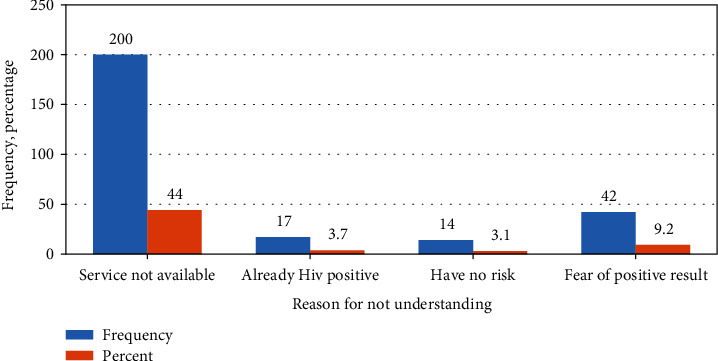
Reason of not undergoing HIV testing among mining workers in Dima district Gambella region southwest Ethiopia, November 2019.

The leading reason listed by mining workers for not undergoing regular HIV testing was service is not easily available (Figure 3).

### 3.7. Attitude towards HIV Prevention

**Figure 4:**
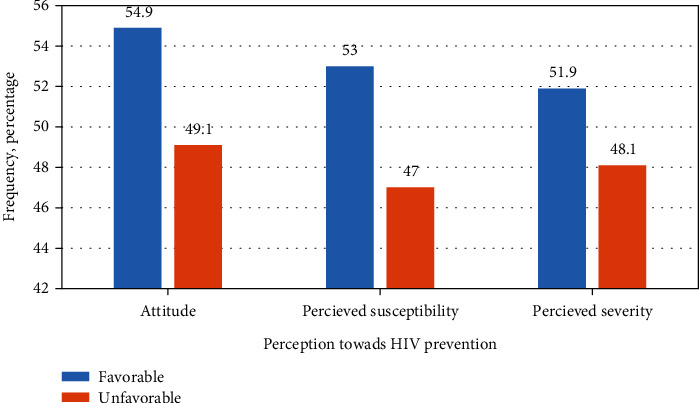
Perceptions towards HIV prevention among mining workers in Dima district Gambella region southwest Ethiopia, November 2019.

From total respondents, 250 (54.9%) had a favorable attitude towards HIV prevention while 205 otherwise. Regarding susceptibility for HIV, 241 (53%) of the respondents had high perceived susceptibility for HIV (Figure 4).

### 3.8. Factors Associated with the Practice of HIV Preventive Behaviors

To control confounding factors, multivariable binary logistic regression was conducted and the number of factors was identified as predictors of good practice of HIV prevention. Among those identified factors, alcohol drinking is the one. Mining workers who do not drink alcohol were 2.86 times likely to practice preventive behaviors than those who drink alcohol (AOR = 2.86, 95% CI: 1.30-6.29). The respondents who do not chew khat were 2.09 times more likely to practice preventive behaviors than those who chew khat (AOR = 2.09, 95% CI: 1.09-4.02). Also, mining workers that had good comprehensive knowledge about HIV were 1.81 times more likely to practice preventive behaviors than their counterparts (*AOR* = 1.81, CI: 1.16-2.83). Mining workers that had favorable attitude towards HIV prevention were 4.76 times more likely to practice preventive behaviors than those with a negative attitude (AOR = 4.76, 95% CI: 3.02-7.49). Lastly, respondents who had high perceived susceptibility for HIV were 2.63 more likely to practice preventive behaviors than respondents with low perceived susceptibility (AOR = 2.63, 95% CI: 1.63-4.24) (Table 4).

## 4. Discussion

This study is aimed at measuring the magnitude of HIV preventive behaviors among mining workers. So, the study showed that 61.3% of respondents had good HIV preventive behaviors. The finding is higher than the study conducted among mining workers in Sali traditional gold mining site bench Maji zone, southwest Ethiopia, which showed only 47.6% of participants were engaged in HIV preventive behavior [28]. This discrepancy might be because of the study conducted among Sali traditional gold mining being late when compared to the current study. Additionally, there are many nongovernmental organizations working on HIV prevention in Gambella region (current study area) than Sali, Bench Maji zone.

According to this study, mining workers that do not drink alcohol were more likely to practice HIV preventive behaviors than those drinking alcohol. Similarly, a study conducted on the effects of hazardous and harmful alcohol use on HIV incidence and sexual behavior showed that unsafe sex, partner violence, and HIV incidence were higher in women with alcohol users [29]. The study conducted among college students in Gambella town also showed that alcohol drinkers are less likely to practice preventive behaviors than nonalcohol drinkers [24]. Similarly, a study conducted on alcohol use and HIV risk behaviors among rural adolescents in Khanh Hoa Province, Vietnam, showed that alcohol use was significantly associated with engagement in sexual behaviors [30]. Also, a study conducted in Jigawa State, Nigeria, indicated that HIV/AIDS knowledge was positively correlated with HIV preventive behaviors [31]. This might be because workers who do not drink alcohol can control his emotions and activities since his mind is relatively stable. In another way, workers drinking alcohol can be influenced by others because of peer pressure and the effect of alcohol on their control.

Similarly, mining workers who do not chew chat were more likely to practice HIV preventive behaviors than chat chewers. Similarly, a study conducted in Northwest and western Ethiopia revealed that chewing khat is associated with having risky sexual behaviors [32, 33].

Individuals who have comprehensive knowledge about HIV were more likely to practice HIV preventive behaviors than an individual lacking comprehensive knowledge about the disease. A study from Lao PDR on risk perceptions of STIs/HIV and sexual risk behaviors among sexually experienced adolescents showed that participants with good knowledge have risk perception of STIs/HIV which leads them to better prevention [34]. In addition to this, a study conducted among female sex workers in Padang revealed that respondents who had good knowledge of HIV/AIDS have better preventive behavior [35].

Similarly, a study conducted on HIV/AIDS preventive behavior among college students in Gambella town, southwest Ethiopia, using the health belief model, indicated that participants with good knowledge were more likely to practice HIV preventive behaviors than those with poor knowledge [24]. This might be because individuals with comprehensive knowledge protect themselves since they know how HIV can be transmitted and the effect of HIV on their life.

Also, mining workers who have a favorable attitude about HIV prevention were more likely to practice HIV preventive behaviors than workers with an unfavorable attitude. This might be because those individuals with favorable attitudes may have knowledge about the disease and can easily protect themselves from getting the disease. This is inline with the study conducted in Padang which revealed that respondents who had good attitude towards HIV/AIDS have better preventive behavior [35].

In this study, perceived susceptibility of the workers toward HIV determines their practice of HIV prevention. Participants who had high perceived susceptibility for HIV were more likely to practice preventive behaviors than respondents with low perception. Similarly, a study conducted on the utilization of HIV/AIDS prevention methods among university students residing at a selected university campus showed that perceived susceptibility to HIV/AIDS showed a correlation with self-efficacy on condoms and their utilization [36]. This is because when they know as they are susceptible, they can take preventive measures.

### 4.1. Limitation of the Study

Social desirability bias may affect this study since the participants might hide some practices. Also, the nature of a cross-sectional study does not show causalities.

## 5. Conclusion and Recommendation

HIV preventive behavior is low in the study area. HIV preventive behaviors were associated with alcohol drinking, chat chewing, knowledge related to HIV, attitude towards HIV prevention, and perceived susceptibility for HIV. Increasing behavior of HIV preventive behaviours through behavioural change connunication is the key intervention. Ethiopia should develop HIV/AIDS policy for the mining sector.

## Figures and Tables

**Table 1:** Sociodemographic characteristics among mining workers in Dima district Gambella region southwest Ethiopia, November 2019.

Variables	Frequency	Percent
*Age group*		
<20	26	5.7
20-24	173	38.0
25-34	220	48.4
>35	36	7.9
*Marital status*		
Single	300	65.9
Married	95	20.9
Divorced/widowed	60	13.2
*Religion*		
Orthodox	274	60.8
Muslim	90	20.0
Protestant	85	18.8
Ethnicity		
Bench	37	8.1
Meinit	30	6.6
Amhara	126	27.7
Wolayita	89	19.6
Oromo	62	13.6
Kafa	93	20.4
Other^∗^	18	4.0
*Educational status*		
No education	47	10.3
Primary	223	49.0
Secondary and above	185	40.7
*Income*		
Less than 1000	256	56.3
1000 and above	199	43.7

^∗^Tigre, Gurhaghe, Hadiya, and Sheko.

**Table 2:** The frequency of correctly answered regarding knowledge about HIV prevention and the perception of HIV among mining workers in Dima district Gambella region southwest Ethiopia November 2019.

	Variable	Frequency (%)
HIV prevention measures	Abstinence	428 (94.1)
	Being faithful	346 (76.0)
	Consistent condom use	252 (55.4)
	Not sharing sharp materials	203 (44.6)
	Getting health information	143 (31.4)
	Treating STI	67 (14.7)
	Male circumcision	62 (13.6)
	PMTCT	121 (26.6)
	ART for exposed individuals	60 (13.2)
	ART for HIV patients	59 (13.0)

Perception of HIV	Withdrawal of the penis before ejaculation prevents HIV transmission	124 (27.3)
	A woman can get HIV if she has anal sex with a man	195 (42.9)
	Washing genitals after prevents HIV transmission	107 (23.5)
	There is a vaccine that for HIV	82 (18.2)
	People are likely to get HIV by kissing	273 (60.2)
	HIV can be transmitted through sharing meals	97 (21.3)
	HIV can be transmitted through the mosquito bite	107 (23.5)

**Table 3:** Practices of preventive behavior among mining workers in Dima district Gambella region southwest Ethiopia, November 2019.

Variables	Frequency (%)
*Ever sexual intercourse*	
Yes	415 (91.2)
No	40 (8.8)
*Sexual activity in the last 12 months*	
Yes	389 (85.5)
No	22 (4.8)
*Sexual partners*	
Regular partner	89 (21.7)
Casual partner	235 (57.3)
Commercial sex worker	82 (20.0)
*Number of sexual partners*	
One	97 (30.5)
Two and above	221 (69.5)
*Condom use on last sexual intercourse*	
Yes	337 (90.8)
No	34 (9.2)
*The habit of condom use*	
Consistently	282 (81.3)
Sometimes	53 (15.3)
Rarely	10 (2.9)
Occasionally	2 (0.6)
*HIV test with in the last three months*	
Yes	182 (40.0)
No	273 (60.0)
*Shared sharp materials in the three months*	
Yes	107 (23.5)
No	348 (76.5)

**Table 4:** Factors associated with HIV preventive behaviors among mining workers in Dima district Gambella region southwest Ethiopia, November 2019.

Variables	Preventive behaviors		COR	AOR
	Yes	No		
*Age group*				
Less than 26	155	101	1	1
26 and above	124	75	1.08 (0.74-1.58)	1.55 (0.96-2.50)
*Educational status*				
No education	28	19	1	
Primary	123	100	0.84 (0.44-1.58)	0.56 (0.27-1.17)
Secondary and above	128	57	1.52 (0.79-2.95)	1.10 (0.51-2.34)
*Alcohol drinking*				
Yes	224	160	1	1
No	55	16	2.46 (1.36-4.44)	2.86 (1.30-6.29)^∗^
*Khat chewing*				
Yes	195	146	1	
No	80	30	1.20 (1.25-3.20)	2.09 (1.09-4.02)^∗^
*Smoking cigarette*				
Yes	112	97	1	1
No	167	79	1.83 (1.25-2.68)	1.44 (0.91-2.27)
*Comprehensive knowledge of HIV*				
Poor	122	105	1	1
Good	156	70	1.92 (1.31-2.82)	1.81 (1.16-2.83)^∗^
*Attitude towards HIV prevention*				
Negative	92	113	1	1
Positive	187	63	3.65 (2.45-5.42)	4.76 (3.02-7.49)^∗^
*Perceived susceptibility for HIV*				
Low	111	103	1	1
High	168	73	2.14 (1.46-3.14)	2.63 (1.63-4.24)^∗^
*Perceived severity of HIV*				
Low	115	103	1	1
High	162	73	1.99 (1.36-2.92)	1.50 (0.93-2.40)

## Data Availability

All data generated during and/or analyzed during the study are available from the corresponding author on reasonable request.

## Abbreviations

AIDS: Acquired immune deficiency syndrome
AOR: Adjusted odds ratio
ART: Antiretroviral therapy
COR: Crude odds ratio
HIV: Human immune deficiency virus
PMTCT: Prevention of mother to child transmission
STI: Sexually transmitted infection.
